# Covalent Bonding of Si Nanoparticles on Graphite Nanosheets as Anodes for Lithium-Ion Batteries Using Diazonium Chemistry

**DOI:** 10.3390/nano9121741

**Published:** 2019-12-06

**Authors:** Yi Zhang, Jinghui Ren, Tao Xu, Ailing Feng, Kai Hu, Nengfei Yu, Yingbin Xia, Yusong Zhu, Zhengyong Huang, Guanglei Wu

**Affiliations:** 1 School of Energy Sciences and Engineering, Nanjing Tech University, Nanjing 211816, Chinazyx7452@njtech.edu.cn (T.X.); 201861108005@njtech.edu.cn (K.H.); yunf@njtech.edu.cn (N.Y.); leslie@njtech.edu.cn (Y.X.);; 2 Institute of Physics & Optoelectronics Technology, Baoji University of Arts and Sciences, Baoji 721016, China; ailing@mail.xjtu.edu.cn; 3 State Key Laboratory of Power Transmission Equipment & System Security and New Technology, Chongqing University, Chongqing 400040, China; 4 Institute of Materials for Energy and Environment, State Key Laboratory of Bio-fibers and Eco-textiles, College of Materials Science and Engineering, Qingdao University, Qingdao 266071, China; 5 Key Laboratory of Engineering Dielectrics and Its Application, Ministry of Education, Harbin University of Science and Technology, Harbin 150080, China

**Keywords:** silicon, graphite nanosheets, diazotization reaction, anode

## Abstract

Silicon/carbon (Si/C) composite has been proven to be an effective method of enhancing the electrochemical performance of Si-based anodes for lithium-ion batteries (LIBs). However, the practical application of Si/C materials in LIBs is difficult because of the weak interaction between Si and C. In this study, we applied two-step diazotization reactions to modify graphite nanosheets (GNs) and Si nanoparticles (Si NPs), yielding a stable Si–Ar–GNs composite. Owing to aryl (Ar) group bonding, Si NPs were dispersed well on the GNs. The as-prepared Si–Ar–GNs composite delivered an initial reversible capacity of 1174.7 mAh·g^−1^ at a current density of 100 mAh·g^−1^. Moreover, capacity remained at 727.3 mAh·g^−1^ after 100 cycles, showing improved cycling performance. This synthesis strategy can be extended to prepare other Si/C anode materials of LIBs.

## 1. Introduction

Lithium-ion batteries (LIBs) are the energy storage devices that drive today’s global portable electronic device market and show great potential for wider applications such as electric vehicles (EVs) because of their high specific energy, long cycle life, lightweight properties, and environmental friendliness [1,2,3,4,5]. However, current LIBs cannot keep pace with the increasing power demands for EVs [6]. LIBs have an anode and a cathode; the two materials exchange lithium ions, which is the key to improving the performance of LIBs. In order to improve the energy density of LIBs, new anode materials must be investigated.

Many groups have spent decades working to develop novel anode materials for LIBs [7,8,9,10,11,12]. Among these materials, silicon holds great potential as an anode material due to its high theoretical gravimetric capacity of 4200 mAh·g^−1^, compared with graphite’s 372 mAh·g^−1^, because it is great insert/de-insert many more lithium ions than the graphite in current LIBs. However, after a few charge-and-discharge cycles, silicon is prone to fracturing and breaking due to its expansion and contraction during de-alloying/alloying processes [13,14,15,16,17]. These issues can be addressed by designing novel anodes incorporating Si and C, such as Si/amorphous carbon [18,19], Si/carbon nanotubes [20,21], and Si/graphene composite anodes [22,23], which may offer a high electrical conductivity and a good mechanical flexibility to accommodate Si volume expansion during the lithiation process.

Graphite nanosheets (GNs) are a kind of two-dimensional (2D) carbon material. Because of their excellent electrical conductivity, GNs have been widely used as electrical conductive filler [24,25]. In our previous papers, we prepared Ag/GNs [26] and Ni/GNs composites [27]. We found that GNs have a high specific surface that is beneficial for the deposition of Si nanoparticles (Si NPs). Thus, GNs have great potential to provide a better cycle life for Si/C anodes because of their unique structure and high electrical conductivity. However, the basal plane *sp*^2^ carbon atoms of GNs and Si NPs are weak. It is difficult to deposit Si NPs on the surface of GNs. Diazonium chemistry is an effective method to modify the surface of GNs and improve the binding of GNs and nanoparticles [28]. *p*-phenylenediamine contains two NH_2_ groups that can result in two diazotization reactions. Thus, we covalented modified GNs with *p*-phenylenediamine, forming NH_2_–Ar–GNs by diazotization reaction. The resulting NH_2_–Ar–GNs reacted with Si to yield a stable Si–Ar–GNs composite by another step diazotization reaction. Owing to Ar group bonding, the as-prepared Si–Ar–GNs composite anode showed better cycling stability compared with the bare Si anode. 

## 2. Experimental Section

### 2.1. Materials

The natural graphite powders (400 mesh) were purchased from Nanjing XFNANO Materials Tech Co., Ltd. (Nanjing, China). Si NPs (200 nm) were purchased from Sigma-Aldrich (St. Louis, MO, USA). Other chemicals were provided by Sinopharm Chemical Reagent Co., Ltd. (Shanghai, China).

### 2.2. Synthesis of Si–Ar–GNs Composite

**Figure 1:**
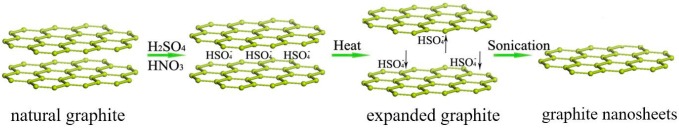
The schematic illustration of graphite nanosheets (GNs) preparation.

The GNs were prepared as follows. The natural graphite powders were added to a mixed acid containing nitric acid and sulfuric acid (1:4, *v*/*v*) at 25 °C for one day. The acidized graphites were heated at 1050 °C for 12 s in a muffle furnace to prepare expanded graphite. The expanded graphite was stirred in an 80% aqueous alcohol solution at 25 °C for one day. Then the mixture was sonicated to break expanded graphite into nanoscale sheets. Finally, the resulting dispersion was filtered, washed, and dried in a freezing vacuum dryer to yield GNs. The schematic illustration of the synthesis of GNs is illustrated in Figure 1.

**Figure 2:**
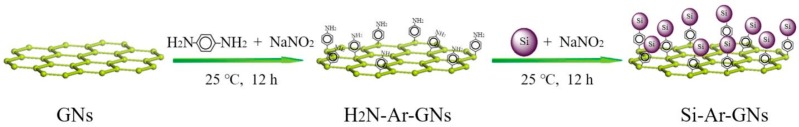
The schematic illustration of Si–Ar–GNs preparation.

A total of 0.1 g of GNs was immersed in 100 mL of acetonitrile containing 21 mmol *p*-phenylenediamine, followed by adding 21 mmol sodium nitrite. The mixture was stirred for 12 h at 25 °C. Then, the mixture was filtered, washed, and dried at 70 °C for 12 h to obtain NH_2_–Ar–GNs. The obtained NH_2_–Ar–GNs were dispersed in 100 mL of acetonitrile, followed by adding 0.05 g of Si NPs. Then, 21 mmol of sodium nitrite was added. The mixture reacted for 12 h at 25 °C. After being filtered, washed, and dried at 70 °C for 12 h, a Si–Ar–GNs composite was obtained. A schematic illustration of the synthesis of the Si–Ar–GNs composite is illustrated in Figure 2. For comparison, Si NPs and GNs (m (Si)/m (GNs) = 1:2) were ball milled to prepare a Si NPs mixed with GNs (Si/GNs) composite.

### 2.3. Characterization 

The phase compositions of GNs were analyzed by X-ray powder diffraction (XRD, Rint-2000, Rigaku) with Cu Kα radiation. Raman spectra were taken by a Reinishaw Raman (RE01) equipped with a 514 nm diode laser. FTIR analyses were conducted using a Bruker Vertex 70 spectrometer from 750 to 4250 cm^−1^ at a resolution of 4 cm^−1^. The morphologies of the Si–Ar–GNs composite and the Si/GNs composite were observed by scanning electron microscopy (SEM, Quanta 250, FEI Inc., Hillsboro, OR, USA). The internal morphologies of the Si–Ar–GNs composite and the Si/GNs composite were studied using a transmission electron microscope (TEM, JEOL, JEM-2100 HT) and HRTEM.

### 2.4. Electrochemical Characterization

Homogeneous slurry was prepared by mixing 80 wt % of Si–Ar–GNs composite, 10 wt % of Super-P carbon black, and 10 wt % of polyacrylic acid (PAA) binder in deionized water. The slurry was then applied to Cu foil as a current collector and dried in a vacuum oven at 100 °C for 24.0 h. The thickness of the electrodes was 70 μm. Coin-type 2025 half-cells were fabricated in an argon-filled glove box (Lab2000, Etelux, China) using the coated Cu disc as the working electrode, lithium foil as the counter electrode, 1 M LiPF_6_ in a mixture of diethyl carbonate (DEC) and ethylene carbonate (EC) (1:1, *v*/*v*) as the electrolyte, and Celgard 2320 membrane as the separator. The galvanostatic charge-discharge performance was tested on a battery test system (BT2000, Arbin, USA) from 0.01 to 3.0 V. Cyclic voltammetry (CV) property was tested by an electrochemical station (PGSTAT 302N, Metrohm, Switzerland) in the 0.01–2.0 V window at a scan rate of 0.2 mV s^−1^.

## 3. Results and Discussion

**Figure 3:**
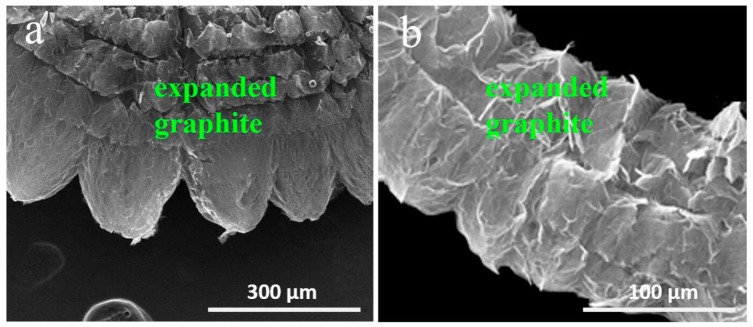
SEM images of expanded graphite: (**a**) low magnification; (**b**) high magnification.

To investigate the morphology of the expanded graphite, SEM analysis was carried out. Figure 3 shows SEM images of expanded graphite under different magnifications. Figure 3a shows that the expanded graphite displayed a worm-like morphology. Figure 3b indicates that expanded graphite showed a loosely packed structure that contained multilayered graphite sheets of nanoscale thickness and microscale diameter. Expanded graphite was prepared by the intensive heating of H_2_SO_4_–graphite intercalation composite. During the synthesis, huge energy led to H_2_SO_4_ decomposition that resulted in a high expansion of graphite. Thus, the expanded graphite formed a loose and porous structure with a high surface area.

**Figure 4:**
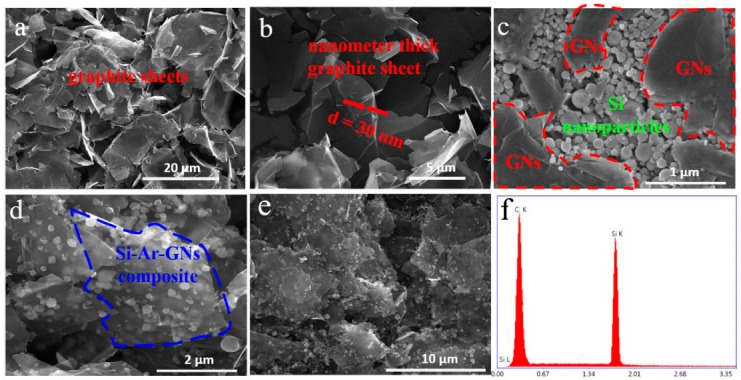
(**a**,**b**) SEM images of graphite nanosheets (GNs); (**c**) SEM image of Si/GNs; (**d**,**e**) SEM images of Si–Ar–GNs; (**f**) energy dispersive X-ray (EDX) spectrum of the Si–Ar–GNs composite.

Figure 4 displays SEM images of GNs, Si/GNs composites, and Si–Ar–GNs composites. GNs were made by sonicating the expanded graphite in an aqueous alcohol solution. After sonication, the expanded graphite was peeled to nanoscale sheets with a diameter range of 10–20 μm (Figure 4a). Figure 4b shows that the GNs were quite thin, and the thickness was around 30 nm. The surface area of the GNs was 101.2 m^2^·g^−1^, suggesting a high specific surface for Si nanoparticle dispersion. However, the surface carbon atoms of graphite have a weak interaction with particles. It was difficult to deposit Si NPs on the GNs’ surface. Figure 4c shows most of the Si NPs of Si/GNs agglomerated in edge areas of GNs. Thus, the surface chemistry of CNs played a vital role in depositing Si NPs. The surface chemistry of GNs and Si NPs yielded a strong chemical bond (phenyl group) between the GNs and Si NPs, forming a uniformly dispersed structure (Figure 4d). To test each component’s content of Si–Ar–GNs composite, energy dispersive X-ray (EDX) analyses were conducted. Figure 4e,f shows the SEM and EDX spectrum of the Si–Ar–GNs composite. Two strong peaks were assigned to C and Si. The composition of Si–Ar–GNs composite calculated from EDX analysis was 67.3 wt % C and 32.7 wt % Si.

**Figure 5:**
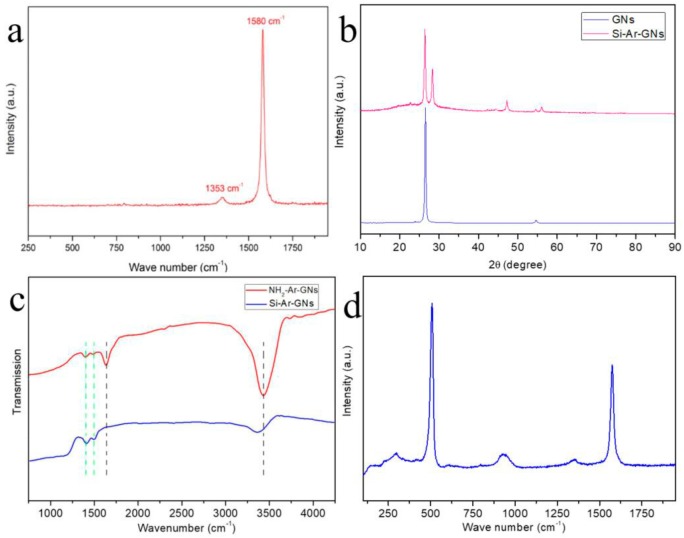
(**a**) Raman spectrum of GNs; (**b**) XRD patterns of GNs and Si–Ar–GNs; (**c**) FTIR spectrum of H_2_N–Ar–GNs and Si–Ar–GNs; (**d**) Raman spectrum of Si–Ar–GNs.

The crystal structure of the GNs was identified using a Raman spectrum as shown in Figure 5a. The peaks in the Raman spectrum at 1345 cm^−1^ and 1575 cm^−1^ corresponded to the D-band (defect) and G-band, respectively. The G-band originated from graphitic structures (*sp*^2^), and the D-band arose from *sp*^3^ carbon and was sensitive to carbon impurities. 

The ratio of the integrated area of the D-band to the G-band, R (*I*_D_/*I*_G_), indicated the degree of carbonization [29,30]. A low R value means a high purity of graphite. The calculated R value of the GNs was 0.04, reflecting a high degree of graphitization. Figure 5b shows the XRD patterns of the GNs and Si–Ar–GNs. The diffraction peaks at 26.4° and 54.4° can be indexed as the (002) and (004) planes of graphite (JCPDS No. 41-1487) [31], respectively. The peaks at about 28.3°, 47.4°, and 56.2° were assigned to (111), (220), and (311) planes of cubic phase silicon (JCPDS No. 27-1402) [32], respectively. The Si–Ar–GNs was characterized by FTIR spectroscopy (Figure 5c). The FTIR spectrum of NH_2_–Ar–GNs showed peaks at around 3450, 1635, 1489, and 1395 cm^−1^. The peaks at 3450 and 1635 cm^−1^ belonged to N–H stretching and bending vibration, while peaks at around 1489 and 1395 cm^−1^ were assigned to C=C and C–H stretching vibration [33], respectively, indicating that NH_2_–Ar was grafted onto the GNs’ surface. After reacting with Si NPs, the peaks belonging to phenyl retained while peaks assigned to NH_2_ disappeared, which revealed that Si NPs were successfully linked to the GNs’ surface via a phenyl group. Moreover, a new weak peak at 3440 cm^−1^ appeared that was assigned to the stretching mode of the Si–OH bonds. Figure 5d shows the Raman spectrum of Si–Ar–GNs. The peaks in the Raman spectrum at 509 cm^−1^ and 930 cm^−1^ were typical features of the Si vibration mode [34]. The ratio of the integrated area of D-band to G-band was still low, which meant the surface chemistry of the GNs did not affect the degree of graphitization. 

**Figure 6:**
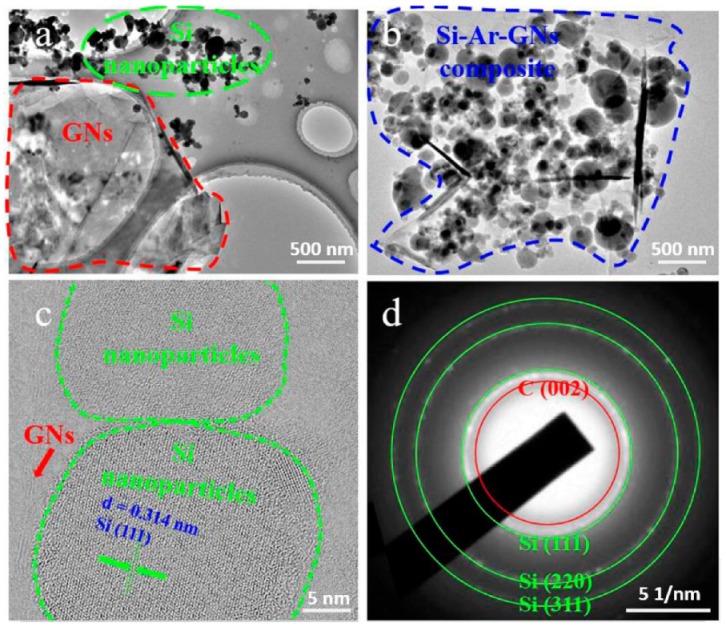
(**a**) TEM image of a Si/GNs composite; (**b**) TEM image of a Si–Ar–GNs composite; (**c**) HRTEM image of a Si–Ar–GNs composite; (**d**) SAED pattern of Si–Ar–GNs composite.

Figure 6 shows TEM, HRTEM, and SAED of Si/GNs and Si–Ar–GNs composites. The agglomeration of Si NPs existed in Si/GNs composites (Figure 6a). For Si–Ar–GNs composites, Si NPs dispersed well on the GNs’ surface (Figure 6b). The HRTEM in Figure 6c clearly displayed high crystallinity of Si NPs with a lattice spacing of 0.314 nm, corresponding to a (111) plane of Si [35]. The few-layered structure of graphite can also be clearly observed in the HRTEM. The diffraction in the SAED pattern (Figure 6d) was assigned to the crystal planes of C (002) and Si (111), Si (220), Si (311). All these results agreed well with the SEM images, demonstrating that the Si NPs were strongly fixed on the GNs’ surface by phenyl groups.

**Figure 7:**
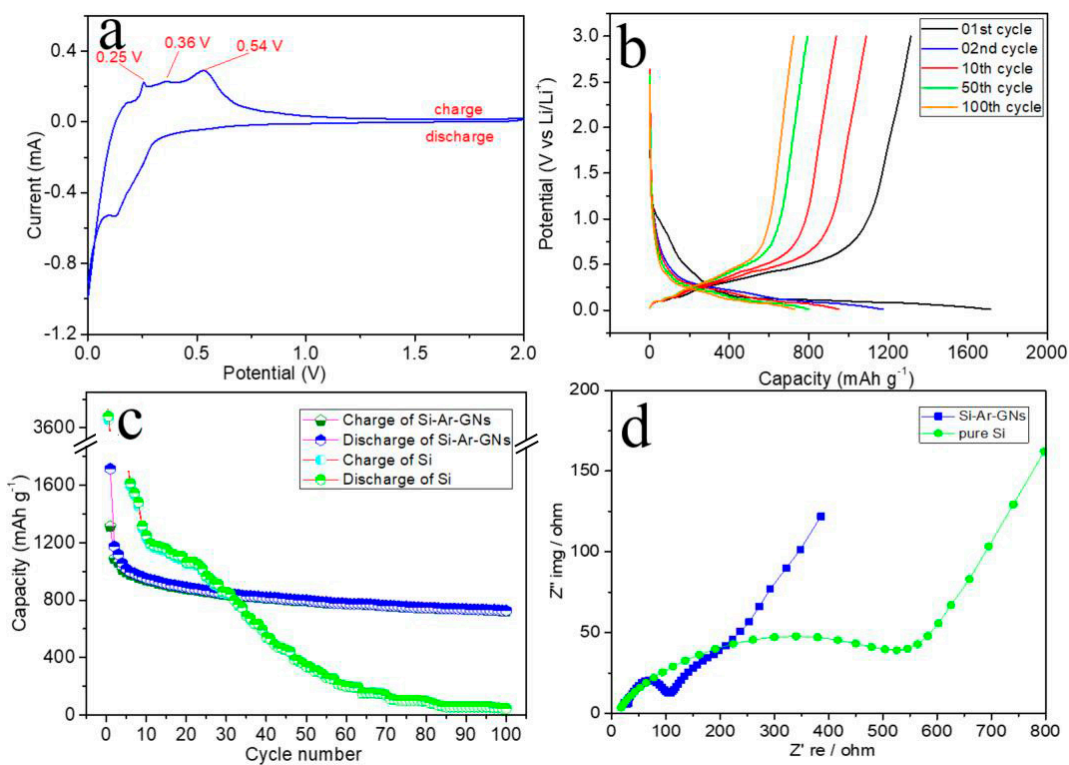
(**a**) Cyclic voltammetry (CV) of the Si–Ar–GNs composite electrode at the scan rate of 0.2 mV·s^−1^; (**b**) galvanostatic charge/discharge profiles of the Si–Ar–GNs composite; (**c**) cycling performance of pure Si and Si–Ar–GNs composites; (**d**) electrochemical impedance spectroscopy (EIS) of the Si–Ar–GNs composite and pure Si.

The electrochemical performance of Si–Ar–GNs is shown in Figure 7. Figure 7a shows a cyclic voltammetry (CV) curve of a Si–Ar–GNs composite at the second cycle. The CV curve clearly shows that some peaks appeared at 0.25, 0.36, and 0.54 V during charge. The peak at 0.25 V was due to the formation of LiC_x_ [36]. The peaks at 0.36 and 0.54 V corresponded to the formation of LixSi [37]. Figure 7b shows the charge-discharge curves of the 1st, 2nd, 10th, 20th, 50th, and 100th cycles of Si–Ar–GNs composite electrodes in 0.01–3.0 V at 100 mA·g^−1^, respectively. The discharge curves had a voltage plateau around 0.3 V, corresponding to a reversible reaction from Si to LixSi [38]. The first discharge and charge capacities of the Si–Ar–GNs composite were 1715.2 mAh·g^−1^ and 1313.3 mAh·g^−1^ with a Coulombic efficiency in the first cycle of 76.6%. The irreversible capacity in the first cycle was due to the formation of SEI film. In later cycles the coulombic efficiency rose to above 99.0%, which indicated a good reversibility of the Si–Ar–GNs composite. Figure 7c displays the cycling performance of the Si–Ar–GNs composite and pure Si anode at 100 mA·g^−1^. Pure silicon exhibited a high first reversible capacity of 3132.9 mAh·g^−1^. However, its capacity quickly decreased to 45.4 mAh·g^−1^ after 100 cycles. The initial reversible capacity of the Si–Ar–GNs composite was 1174.7 mAh·g^−1^ and maintained at 727.3 mAh·g^−1^ at the 100th cycle, corresponding to a capacity retention of 61.9%, which was much higher than that of pure Si (only 1.4%). Moreover, this result was higher than that of other similar Si/C anodes [39,40,41]. The improved cycle performance could be ascribed to the strong bonding between Si NPs and CNs, which can effectively buffer the huge volume change. This improvement was studied by electrochemical impedance spectroscopy (EIS) measurements (Figure 7d) on the Si–Ar–GNs composite and pure Si cells. The semicircle on the EIS spectrum of the Si–Ar–GNs composite was smaller than that on the spectrum of pure Si, indicating that the Si–Ar–GNs composite had a lower charge transfer resistance.

**Figure 8:**
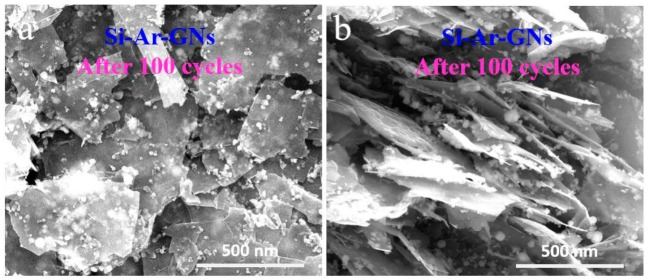
SEM images of the Si–Ar–GNs composite after 100 cycles: (**a**) top view; (**b**) side view.

The improvement of the capacity retention of the Si–Ar–GNs composite could be ascribed to the following effects. First, owing to Ar group bonding, Si NPs can disperse well on GNs and avoid aggregation during cycling processes. To study this banding effect, the morphologies of the Si–Ar–GNs composite after 100 cycles was examined. SEM images (Figure 8a,b) show that Si NPs still remain on the GNs’ surface without aggregation, which means that the surface chemistry of the GNs and Si NPs was effective. Second, GNs have good mechanical flexibility that can accommodate the volume expansion of Si during lithiation process. Third, GNs with high electrical conductivity offer an effective electron transfer path, which could promote electron transfer during the lithiation and delithiation processes. 

## 4. Conclusions 

In summary, Si–Ar–GNs composites were successfully prepared via a two-step diazotization reaction. This method made use of the chemical functions of GNs and Si NPs to yield a covalent bond (Ar group) between GNs and Si NPs. Thus, Si NPs were uniformly coated on the GNs’ surface. The covalent bond avoided Si NPs aggregation during cycling processes, and the GNs accommodated the large volume changes of Si NPs, thus improving the cycling stability of the anode. The Si–Ar–GNs composite delivered an initial reversible capacity of 1174.7 mAh·g^−1^ at a current density of 100 mA·g^−1^. A capacity of 727.3 mAh·g^−1^ was obtained at 100 mA·g^−1^ for 100 cycles, which was higher than that of pure Si (only 45.4 mAh·g^−1^) and other similar Si/C anodes. The electrochemical performances suggest that the Si–Ar–GNs composite is an ideal anode material for LIBs. Our work provides a simple surface chemistry method to improve the cycling performance of Si/C anode materials.
